# Red blood cell transfusion is an independent risk factor for cardiovascular complications in adult patients undergoing cardiac surgery: a propensity score-matched analysis

**DOI:** 10.1186/cc10213

**Published:** 2011-06-22

**Authors:** JP Almeida, F Galas, JL Vincent, JT Fukushima, RE Nakamura, R Kalil Filho, FB Jatene, JOC Auler, LA Hajjar

**Affiliations:** 1 InCor, São Paulo - SP, Brazil

## Introduction

Red blood cell (RBC) transfusion is associated with a higher occurrence of clinical complications after cardiac surgery. However, the cause-effect relationship is confounded by other risk factors for worse outcomes as advanced age, valve or combined procedure, high EuroSCORE, redo surgery, longer bypass time and previous anemia. The objective of this study was to evaluate the effect of RBC transfusion in a propensity score-matched case-control analysis.

## Methods

A total of 502 patients who underwent cardiac surgery with cardiopulmonary bypass from February 2009 to February 2010 were evaluated. We performed a propensity score-matching analysis in 264 patients, considering the following risk factors for cardiovascular complications: sex, age, type of procedure, EuroSCORE, redo surgery, bypass time and previous hemoglobin.

## Results

Cardiovascular complications occurred in 39 patients (30%) exposed to red blood cell transfusion, and in 22 patients (17%) not exposed. The propensity score-matched analysis showed an odds ratio of 2.1 (95% CI = 1.2 to 3.8) for cardiovascular complications in patients exposed to RBC transfusion (Table 1).

**Table 1:** Comparison between propensity-matched patients groups with and without red blood cell transfusion after cardiac surgery

	RBC transfusion		
		
Variable	No (*n *= 132)	Yes (*n *= 132)	*P *value
Sex (female)	34 (26%)	48 (36%)	0.063
Age (years), mean (95% CI)	59 (57 to 61)	60 (58 to 63)	0.295
Procedure			
CABG	89 (67%)	87 (66%)	0.873
Valve	36 (27%)	36 (27%)	
CABG + valve	7 (5%)	9 (7%)	
EuroSCORE, median (IQR)	4 (3 to 6)	4 (3 to 6)	0.683
Redo surgery	13 (10%)	14 (11%)	0.839
Bypass time (minutes), median (IQR)	89 (75 to 110)	91 (75 to 112)	0.418
Hemoglobin (g/dl), mean (95% CI)	13.3 (13.1 to 13.6)	13.1 (12.9 to 13.3)	0.192
Cardiovascular complications	22 (17%)	39 (30%)	0.013
	OR = 2.1 (95% CI = 1.2 to 3.8)		

CABG, coronary artery bypass surgery; CI, confidence interval; IQR, interquartile range; OR, odds ratio. Statistical tests: Mann-Whitney and chi-square.

## Conclusion

RBC transfusion after cardiac surgery increases the risk of cardiovascular complications in a group of patients paired for other risk factors. These findings bring into perspective the importance of an adoption of a restrictive strategy of RBC transfusion to avoid cardiovascular complications.

